# Supplementary material for: Analysis of PPARγ Signaling Activity in Psoriasis
Source: Int J Mol Sci. 2021 Aug 10;22(16):8603. doi: 10.3390/ijms22168603 (PMC8395241; doi:10.3390/ijms22168603)
Supplement: Supplementary file 1 [file ijms-22-08603-s001.zip › Supplemental materials_Analysis of PPARg signaling activity in psoriasis/Pathway models/Models images and html files/Model of PPARG signaling in psoriasis/9688.html]

RAC1


# Protein RAC1

|  |  |
| --- | --- |
| URN | urn:agi-llid:5879 |
| Total Entities | 0 |
| Connectivity | 6321 |
| Name | RAC1 |
| Description | ras-related C3 botulinum toxin substrate 1 (rho family, small GTP binding protein Rac1) |
| Notes | The protein encoded by this gene is a GTPase which belongs to the RAS superfamily of small GTP-binding proteins. Members of this superfamily appear to regulate a diverse array of cellular events, including the control of cell growth, cytoskeletal reorganization, and the activation of protein kinases. Two transcript variants encoding different isoforms have been found for this gene. [provided by RefSeq, Mar 2009] |
| Primary Cell Localization | Cytoplasm |
| ObjectType | Protein |

---

|  |  |
| --- | --- |
| Pathway | Oxidative Stress in Amyotrophic Lateral Sclerosis |
|  | Osteoarthritis |
|  | Skin Aging |
|  | Apoptosis of Cochlear Hair Cells (Noise-Induced, Mouse Model) |
|  | Dopamine Receptors Signaling |
|  | Thyroid Hormones Common non-Genomic Effects in Hyperthyroidism |
|  | Toll-like Receptors Act through MYD88-TIRAP Signaling |
|  | T-Cell Dependent B-Cell Activation |
|  | T-Cell Independent B-Cell Activation |
|  | Natural Killer Cell Activation through C-Type Lectin-like Receptors |
|  | Natural Killer Cell Activation through ITAM-Containing Receptors |
|  | Natural Killer Cell Inhibitory Receptor Signaling |
|  | Natural Killer Cell Activation through ITSM-Containing Receptors |
|  | Natural Killer Cell Activation through Integrins and non-ITAM-Containing Receptors |
|  | Natural Killer Cell Activation |
|  | CXC Chemokine Receptor Signaling |
|  | CC Chemokine Receptor Signaling |
|  | Plasmin Effects in Inflammation |
|  | Platelet Activation via Adhesion Molecules |
|  | Scavenger Receptors in Platelet Aggregation |
|  | Bradykinin Effects in Inflammation |
|  | Platelet Activation via GPCR Signaling |
|  | Toll-like Receptors in Sterile Inflammation |
|  | Toll-like Receptor Independent Sterile Inflammation |
|  | Lipoxin A4/FPR2-Related Neutrophil Depression |
|  | Do not Eat me\xc2\x9d Signal: Viable Cells Inhibits Phagocytosis |
|  | Eat me Signal: Apoptotic Cell Induces Phagocytosis |
|  | Neutrophil Activation via LTB4/Polyisoprenyl Phosphate Signaling |
|  | Basophil Activation |
|  | Eosinophil Activation |
|  | Mast-Cell Activation via IgE Signaling |
|  | Mast-Cell Activation without Degranulation |
|  | Mast-Cells De Novo Synthesized Mediators via IgE Independent Signaling |
|  | Mast-Cell Activation without Degranulation through IL1R1 and TLR Signaling |
|  | CD16/CD14 Proinflammatory Monocyte Activation |
|  | Macrophage M2-Related Phagocytosis |
|  | Neutrophil Activation via FCGR3B |
|  | Neutrophil Degranulation via FPR1 Signaling |
|  | CR3 Mediated Phagocytosis in Neutrophils and Macrophages |
|  | Neutrophil Chemotaxis |
|  | Neutrophil Activation via Adherence on Endothelial Cells |
|  | Vascular Smooth Muscle Cell/Pericyte Migration and Proliferation |
|  | Vascular Endothelial Cell Activation by Cytokines |
|  | Vascular Endothelial Cell Permeability Activation |
|  | Dendritic Spine Morphogenesis and Stabilization |
|  | GFs/TNF -> Ion Channels |
|  | DRD2 Expression Targets |
|  | IL1B Expression Targets -> Nociception |
|  | Tight Junction Assembly (Claudins) |
|  | Adherens Junction Assembly (Nectin) |
|  | Adherens Junction Assembly (Cadherins) |
|  | Tight Junction Assembly (JAMs) |
|  | Lamellipodia and Filopodia Formation in Cancer |
|  | Exocytosis: Vesicle Tethering |
|  | Scavenger Receptor OLR1 in Inflammation Related Endothelial Dysfunction |
|  | Proteins Involved in Dilated Cardiomyopathy |
|  | Proteins Involved in Arterial Hypertension |
|  | BMP2 and Non-Canonical WNT Signaling in Pulmonary Artery Endothelial Cells |
|  | Endothelial Cell Dysfunction in Pulmonary Hypertension |
|  | Smooth Muscle Cell Dysfunction in Pulmonary Hypertension |
|  | BMP2 and WNT Signaling in Pulmonary Artery Smooth Muscle Cells |
|  | Myocarditis, Acute Viral Overiew |
|  | Inflammatory Reaction in Acne Vulgaris |
|  | Keratinocyte Proliferation in Acne Vulgaris |
|  | Proteins Involved in Melanoma |
|  | B-Cell Activation in Systemic Lupus Erythematosus |
|  | Neutrophil and Macrophage Function in Systemic Lupus Erythematosus |
|  | Vitiligo |
|  | Apoptosis of Melanocyte in Vitiligo |
|  | Humoral Immunity in Vitiligo |
|  | Natural Killer Cell in Diabetes Mellitus Type 1 |
|  | Macrophage Function in Diabetes Mellitus Type 1 |
|  | Insulin Resistance in Hepatocytes |
|  | beta-Cell Destruction through Cytokines in Diabetes Mellitus |
|  | Myocyte Dysfunction in Hypoparathyroidism |
|  | Hypoparathyroidism, Primary |
|  | Extraocular Muscles Weakness in Myasthenia Gravis |
|  | CD8+ T-Cell Response in Celiac Disease |
|  | CagA Phosphorylation Independent Signaling |
|  | CagA Phosphorylation Dependent Signaling |
|  | Dendritic Cell Function in Ulcerative Colitis |
|  | Nicotine anti-Inflammatory Effect in Ulcerative Colitis |
|  | Antiphospholipid Antibodies in Platelets and Coagulation Cascade |
|  | B-Cell Chronic Lymphocytic Leukemia |
|  | Mucosa-Associated Lymphoid Tissue (MALT) Lymphoma |
|  | Platelet and Coagulation Cascade Activation by Antiphospholipid Antibodies |
|  | Eosinophil Activation and Degranulation in Asthma |
|  | TSLP Signaling in Bronchial Epithelial Cell in Asthma |
|  | Neutrophil Degranulation via FPR1/IL8 |
|  | Basophil Activation in Asthma |
|  | Mast-Cell Activation in Asthma |
|  | Mast-Cell Related Chemokines Signaling in Asthma |
|  | Antigen-Presenting Cell Role in Asthma |
|  | CFTR Facilitates Oxidative Stress in Airway Epithelium in CF |
|  | TNF and IL1B Induce Metalloproteinase Synthesis in Osteoarthritis |
|  | IL1B Induced Arthralgia |
|  | Synovial Fibroblast Activation in Psoriatic Arthritis |
|  | Synovial Fibroblast Activation by Citokines in Rheumatoid Arthritis |
|  | B-Cells Function in Systemic Scleroderma |
|  | CCR5 Signaling in Macrophages |
|  | Macrophage Survival through CCR5 and CXCR4 Mediated Signaling |
|  | IL1 Receptor Antagonist (IL1RN) Deficiency (DIRA) |
|  | Blau Syndrome |
|  | IL36 Receptor Antagonist (IL1F5) Deficiency (DITRA) |
|  | Familial Cold Autoinflammatory Syndrome 2 |
|  | Rett Syndrome |
|  | IL6 Promotes Inflammation in Muscular Dystrophies |
|  | Ca2+ Overload in Muscular Dystrophies |
|  | Muscular Dystrophies |
|  | Dystrophin Glycoprotein Complex Signaling in Muscular Dystrophies |
|  | Proteins Involved in Osteopetrosis |
|  | Proteins Involved in Epilepsy |
|  | Proteins Involved in Glioblastoma |
|  | Proteins Involved in Astrocytoma |
|  | Proteins Involved in Glioma |
|  | Proteins Involved in Medulloblastoma |
|  | Proteins Involved in Neuroblastoma |
|  | SOD1 Mutation |
|  | Endosomal Trafficking Dysregulation in Amyotrophic Lateral Sclerosis |
|  | Microglia and Motor Neuron Interaction Dysregulation |
|  | Proteins Involved in Breast Cancer Related to WNT Signaling Pathway |
|  | WNT Signaling in Breast Cancer |
|  | Angiogenesis in Endometriosis |
|  | Subepithelial Fibroblasts in Middle Ear Cholesteatoma |
|  | VEGFA in Neovascular Glaucoma |
|  | Cytokines Trigger Otitis Media |
|  | Alveolar Epithelial Cell Dysfunction in COPD |
|  | WNT Signaling in Cystic Kidney Disease |
|  | Plasmin Role in Glomerulonephritis |
|  | CD157/ITGB2 Signaling in Myeloid Cell |
|  | CD38/CD3 -> JUN/FOS/NF-kB Signaling in T-cell Proliferation |
|  | CD38/CD19 -> JUN/FOS/NF-kB Signaling in B-cell Proliferation |
|  | S100P Expression Target |
|  | HMGB1 Expression Targets |
|  | TTR Expression Targets |
|  | S100A Expression Targets |
|  | S100B Expression Targets |
|  | ANGPT1 -> CREB/CREBBP Expression Targets |
|  | ANGPT2 -> AP-1/CREBBP/MYC Expression Targets |
|  | AREG -> NFATC Expression Target |
|  | AREG -> CREB/CREBBP Expression Targets |
|  | AREG -> NCOR2 Expression Targets |
|  | AREG -> AP-1 Expression Targets |
|  | Dopamine/Gi Expression Targets |
|  | BTC -> NFATC Expression Targets |
|  | BTC -> AP-1/ATF/CREB Expression Targets |
|  | Frizzled Receptors -> ARRB1/ARRB2 non-Canonical Signaling |
|  | ARRB1/ARRB2 non-CanonicalSignaling and HedgehogFamily |
|  | CD247 Expression Targets |
|  | FCGR3A Expression Targets |
|  | CD72 -> NF-kB Expression Targets |
|  | CD8 -> NF-kB Expression Targets |
|  | CD80 -> NFATC Expression Targets |
|  | CD86 -> NFATC Expression Targets |
|  | EGF -> AP-1/ATF Expression Targets |
|  | EGF -> CREB/CREBBP/ELK/SRF/MYC Expression Targets |
|  | EGF -> MEF/MYOD/NFATC Expression Targets |
|  | EGF -> TP53 Expression Targets |
|  | EGF -> NCOR2 Expression Target |
|  | EGFR -> NCOR2 Signaling |
|  | EGFR/ERBB3 -> MEF/MYOD/NFATC/MYOG Signaling |
|  | EGFR/ERBB2 -> TP53 Signaling |
|  | EGFR -> AP-1/ATF2 Signaling |
|  | EGFR -> AP-1/CREB/ELK/SRF/MYC Signaling |
|  | EREG -> CREB Expression Target |
|  | EREG -> AP-1/ATF Expression Targets |
|  | Erythropoietin -> AP-1/MYC/CREB Expression Targets |
|  | F2 -> AP-1/CREB/ELK/SRF/SP1 Expression Targets |
|  | PLG -> AP-1/CREB/ELK/SRF/SP1 Expression Targets |
|  | FGF9 -> AP-1/CREB/MYC Expression Targets |
|  | FGF10 -> AP-1/CREB/CREBBP/MYC Expression Targets |
|  | FGF1 -> AP-1/CREB/ELK/SRF/MYC Expression Targets |
|  | FGF1 -> NCOR2 Expression Target |
|  | FGF8 -> AP-1/CREB/MYC Expression Targets |
|  | FGF18 -> AP-1/CREB Expression Targets |
|  | FGF23 -> NCOR2 Expression Targets |
|  | FGF2 -> NCOR2 Expression Targets |
|  | FGF4 -> AP-1/MYC Expression Targets |
|  | FGF7 -> AP-1/CREB/CREBBP/MYC Expression Targets |
|  | FGF2 -> AP-1/CREB/CREBBP/ELK/SRF/MYC Expression Targets |
|  | FGFR -> AP-1/CREB/CREBBP/ELK/SRF/MYC Signaling |
|  | FPR1 -> Cytoskeleton Signaling |
|  | FrizzledR -> JUN/PAX2 Signaling |
|  | HBEGF -> AP-1/ATF Expression Targets |
|  | HBEGF -> TP53 Expression Targets |
|  | HBEGF -> MEF/MYOD Expression Target |
|  | HBEGF -> CREB/MYC Expression Targets |
|  | HGF -> AP-1/CREB/ELK/SRF/MYC Expression Targets |
|  | HGFR -> AP-1/CREB/MYC Signaling |
|  | EGFR Transactivation in Cancer and Non-Cancer Cells |
|  | TAM Receptor Signaling Activation in Cancer |
|  | RAS/RAF/MAPK Signaling Activation in Cancer |
|  | STAT3 and NFkB Activate Inflammation-Induced Tumorigenesis |
|  | RAS/RAF/MAPK Signaling Activation by Blocking of Tumor Suppressors |
|  | TGFB Signaling activation by Blocking of Tumor Suppressors |
|  | VEGFA Dependent Angiogenesis in Cancer |
|  | VEGF Independent Angiogenesis in Cancer |
|  | Integrins in Cancer Cell Motility, Invasion and Survival |
|  | Proteins with Altered Expression in Cancer Metastases |
|  | BSG (CD147) in Cancer Cells Motility, Invasion and Survival |
|  | CDH1 Down regulation Promotes Cancer Cell Migration and Metastases |
|  | MTOR/TORC in Cancer Cell Motility and Invasion |
|  | Hyaluronic Acid, CD44 and HMMR in Cancer Cell Invasion and Survival |
|  | CDH2 Activation Promotes Cancer Cell Migration and Survival |
|  | TGFB Family in Epithelial to Mesenchymal Transition in Cancer |
|  | IGF1 -> MEF/MYOD/MYOG Expression Targets |
|  | IGF2/MEF/MYOD Expression Targets |
|  | Insulin -> MEF/MYOD Expression Targets |
|  | AGER -> CREB/SP1 Signaling |
|  | FcIgER -> ELK-SRF Signaling |
|  | IGF1R -> MEF/MYOD/MYOG Signaling |
|  | FibronectinR -> NF-kB Signaling |
|  | FibronectinR -> AP-1/ELK/SRF/SREBF Signaling |
|  | Fibronectin Expression Targets |
|  | CTGF -> AP-1/CREB/MYC Expression Targets |
|  | CTGF -> NCOR2 Expression Target |
|  | IL1R -> STAT3 Signaling |
|  | IL1R -> NF-kB Signaling |
|  | IL16-> ATF/CREB/CREBBP Expression Target |
|  | LPA Expression Targets |
|  | NRG1 -> MEF/MYOD Expression Targets |
|  | NRG1 -> CREB/CREBBP/ELK/SRF/MYC Expression Targets |
|  | NRG1 -> TP53 Expression Target |
|  | NRG1 -> AP-1/ATF Expression Targets |
|  | PDGFB -> AP-1/CREB/MYC Expression Target |
|  | PDGFC -> CREB Expression Target |
|  | PDGFD -> AP-1 Expression Targets |
|  | PDGF -> AP-1/CREB/CREBBP/MYC Expression Targets |
|  | KIT -> MITF Signaling |
|  | PDGFR -> AP-1/MYC Signaling |
|  | AVP/Gs -> CREB/ELK/SRF/AP-1/EGR Expression Targets |
|  | EDN1 Expression Targets |
|  | AVP/Gs -> MEF/MYOD/NFATC/MYOG Expression Targets |
|  | AVP/Gq -> MEF/MYOD/NFATC/MYOG Expression Targets |
|  | EDN3 Expression Targets |
|  | AVP/Gq -> CREB/ELK/SRF/AP-1/EGR Expression Targets |
|  | PDCD1 -> ATF/CREB/CREBBP Expression Targets |
|  | AngiopoietinR -> AP-1 Signaling |
|  | EphrinB -> Cytoskeleton Signaling |
|  | GDNF -> HSF1 Signaling |
|  | EphrinB -> MAPK/JUN/FOS Signaling |
|  | NTRK -> AP-1/CREB/ELK/SRF/MYC/SMAD3/TP53 Signaling |
|  | GAS6 -> AP-1/CREB Expression Targets |
|  | FIGF -> AP-1 Expression Target |
|  | FIGF -> NCOR2 Expression Target |
|  | KITLG -> AP-1/CREB/CREBBP/MYC Expression Targets |
|  | GDNF -> HSF1 Expression Targets |
|  | CSF1 -> AP-1/CREB/CREBBP/MYC Expression Targets |
|  | PGF -> AP-1/CREB/CREBBP/MYC Expression Targets |
|  | FLT3LG -> AP-1/CREB/CREBBP Expression Targets |
|  | Lysophosphatidic Acid/LPARs Signaling |
|  | JNK/MAPK Signaling |
|  | MERTK Signaling |
|  | Ras Signaling |
|  | TGF-beta Signaling |
|  | Ras-GAP Regulation Signaling |
|  | P38 MAPK/MAPK14 Signaling |
|  | AXL Receptor Tyrosine Kinase Signaling |
|  | PI3K/RAC1 Signaling |
|  | VEGF Signaling |
|  | EGFR Signaling |
|  | WNT Planar Cell Polarity (PCP) non-Canonical Signaling |
|  | ERK/MAPK Canonical Signaling |
|  | TCR -> CREB/CREBBP/ATF Expression Targets |
|  | T-Cell Receptor -> ATF/CREB Signaling |
|  | TGFA -> TP53 Expression Targets |
|  | TGFA -> MEF/MYOD/NFATC Expression Targets |
|  | TGFA/AP-1/ATF Expression Targets |
|  | TGFA -> CREB/CREBBP/ELK-SRF/MYC Expression Targets |
|  | Thrombopoietin -> AP-1/CREB/CREBBP/MYC Expression Targets |
|  | TLR1 -> 2/6 Expression Targets |
|  | IL1B Expression Targets |
|  | IL1A Expression Targets |
|  | TLR1/2/6 -> NF-kB Signaling |
|  | ROS in Triggering Vascular Inflammation |
|  | ROS and RNS in Vasoconstriction and Vasodilation Regulation |
|  | ROS in Angiotensin Mediated Cardiovascular Remodeling and Hypertrophy |
|  | VEGFA -> AP-1/CREBBP/MYC Expression Targets |
|  | VEGFA -> NCOR2 Expression Target |
|  | VEGFR -> AP-1/CREB/MYC Signaling |
|  | WNT1 Expression Targets |
|  | WNT3A Expression Targets |
|  | WNT4 Expression Targets |
|  | WNT5A Expression Targets |
|  | WNT7A Expression Targets |
|  | ace2 |
|  | Model of PPARG signaling in psoriasis |
|  | sars-cov no binding |
|  | New Pathway |
|  | 2\_The role of endothelial cells in pyelonephritis |
|  | 3\_Enhanced angiogenesis in endometriosis |
|  | 1\_Disruption of luteinizing hormone and follicle-stimulating hormone secretion in PCOS |
|  | 3\_Arterial wall inflammation and calcification in atherosclerosis |
|  | 2\_Oxidized LDL causes endothelial dysfunction in atherosclerosis |
|  | 1\_Endothelial cells stimulate vasoconstriction and thrombosis in pulmonary hypertension |
|  | 1\_Cigarette smoke causes dysfunction and production of inflammatory mediators in alveolar epithelial cells |
|  | 1\_Th2 cell response in asthma |
|  | 2\_1\_Eosinophilia and neutrophilia in asthma: eosinophil migration and activation |
|  | 2\_2\_Eosinophilia and neutrophilia in asthma: eosinophil apoptosis |
|  | 4\_2\_Airway smooth muscle cells proliferation and airway tissue remodeling |
|  | 2\_Intestine epithelial cells drive immunological responses in ulcerative colitis |
|  | 4\_1\_Polymorphisms associated with inflammatory bowel diseases |
|  | 2\_CD8+ T-cell response and mucosal damage in celiac disease |
|  | 2\_Mutations in SOD1 and associated proteins cause motor neuron death in familial ALS |
|  | 2\_1\_General pathways of thyroid hormone signaling |

---

|  |  |
| --- | --- |
| Group | other partners for SARS |

---

|  |  |
| --- | --- |
| MedScan ID | 5879 |

---

|  |  |
| --- | --- |
| LocusLink ID | 5879 |
|  | 19353 |
|  | 363875 |
|  | 100781 |
|  | 171377 |
|  | 52352 |

---

|  |  |
| --- | --- |
| Alias | p21-Rac1 |
|  | ras-related C3 botulinum toxin substrate 1 isoform Rac1 |
|  | rho family, small GTP binding Rac |
|  | p21-Rac I |
|  | migration-inducing protein 5 |
|  | cell migration-inducing gene 5 protein |
|  | rac1 GTP-binding protein |
|  | ras-related C3 botulinum toxin substrate 1 isoform Rac1b |
|  | ras-related C3 botulinum toxin substrate 1 isoform Rac I |
|  | rho family, small GTP binding protein Rac1 |
|  | rac1c |
|  | ras-related C3 botulinum toxin substrate I |
|  | MGC111543 |
|  | GTP-binding protein rac1 |
|  | small gtp binding protein rac1 |
|  | RAS-related C3 botulinum substrate 1 |
|  | migration-inducing protein V |
|  | MRD48 |
|  | Rac(V12) |
|  | RAS-related C3 botulinum substrate I |
|  | Rac1B |
|  | Rac family small GTPase 1 |
|  | Rac1 |
|  | D5Ertd559e |
|  | rho family, small GTP binding protein Rac I |
|  | DNA segment, Chr 5, ERATO Doi 559, expressed |
|  | LOC136000 |
|  | OTTMUSP00000022620 |
|  | rac1 GTP binding protein |
|  | Ras-like protein TC25 |
|  | ras-related C3 botulinum toxin substrate 1 (rho family small GTP binding protein Rac1) |
|  | ras-related C3 botulinum toxin substrate 1 |
|  | LOC155330 |
|  | GTP-binding protein rac I |
|  | Rac I |
|  | AL023026 |
|  | TC-25 |
|  | RAC1\_HUMAN |
|  | MIG5 |
|  | Rac-1 |
|  | ras-related C3 botulinum toxin substrate 1 (rho family, small GTP binding protein Rac1) |
|  | TC25 |
|  | migration-inducing gene 5 |
|  | Ras-like protein TC25 |
|  | expressed sequence AL023026 |
|  | isoform rac1b |

---

|  |  |
| --- | --- |
| EC Number | 3.6.5.2 |

---

|  |  |
| --- | --- |
| GO ID | 0051117 |
|  | 0005525 |
|  | 0003924 |
|  | 0017137 |
|  | 0051022 |
|  | 0019899 |
|  | 0042826 |
|  | 0019901 |
|  | 0044877 |
|  | 0031996 |
|  | 0038095 |
|  | 0038096 |
|  | 0016601 |
|  | 0007266 |
|  | 0031295 |
|  | 0060071 |
|  | 0030036 |
|  | 0030041 |
|  | 0009653 |
|  | 0007596 |
|  | 0045453 |
|  | 0007155 |
|  | 0016477 |
|  | 0048870 |
|  | 0030031 |
|  | 0008283 |
|  | 0007160 |
|  | 0071260 |
|  | 0048013 |
|  | 0048012 |
|  | 0006954 |
|  | 0035556 |
|  | 0030032 |
|  | 0051668 |
|  | 0002551 |
|  | 0008045 |
|  | 0032707 |
|  | 0048261 |
|  | 0001764 |
|  | 0043312 |
|  | 0045740 |
|  | 0035025 |
|  | 0010811 |
|  | 0051894 |
|  | 0035774 |
|  | 0010592 |
|  | 0031116 |
|  | 0090023 |
|  | 0051897 |
|  | 0001934 |
|  | 0051496 |
|  | 1900026 |
|  | 0030334 |
|  | 0008361 |
|  | 0050690 |
|  | 0010310 |
|  | 0010591 |
|  | 1902622 |
|  | 0045428 |
|  | 0060263 |
|  | 0051056 |
|  | 0051492 |
|  | 0009611 |
|  | 0097178 |
|  | 0031529 |
|  | 0071526 |
|  | 0034446 |
|  | 0048010 |
|  | 0000139 |
|  | 0005884 |
|  | 0042995 |
|  | 0005737 |
|  | 0036464 |
|  | 0031410 |
|  | 0005829 |
|  | 0043197 |
|  | 0005789 |
|  | 0070062 |
|  | 0101003 |
|  | 0005925 |
|  | 0098978 |
|  | 0030027 |
|  | 0042470 |
|  | 0016020 |
|  | 0005886 |
|  | 0098794 |
|  | 0055038 |
|  | 0032587 |
|  | 0030667 |
|  | 0005802 |
|  | 0030742 |
|  | 0000166 |
|  | 0007186 |
|  | 0007015 |
|  | 0048532 |
|  | 0002093 |
|  | 0007411 |
|  | 0045216 |
|  | 0021894 |
|  | 0021799 |
|  | 0006935 |
|  | 0090103 |
|  | 0007010 |
|  | 0016358 |
|  | 0048813 |
|  | 0071542 |
|  | 0021831 |
|  | 0006897 |
|  | 0043652 |
|  | 0003382 |
|  | 0030900 |
|  | 0048873 |
|  | 0006972 |
|  | 1904948 |
|  | 0048812 |
|  | 0035567 |
|  | 0006911 |
|  | 0030838 |
|  | 0060999 |
|  | 0043552 |
|  | 0072659 |
|  | 0022604 |
|  | 0010762 |
|  | 0014041 |
|  | 0048168 |
|  | 0007264 |
|  | 0051932 |
|  | 0031901 |
|  | 0019897 |
|  | 0060091 |
|  | 0005634 |
|  | 0000242 |
|  | 0001891 |
|  | 0008152 |
|  | 0006928 |
|  | 0030168 |
|  | 0043065 |
|  | 0015031 |
|  | 0016023 |
|  | 0005622 |
|  | 0097190 |
|  | 0045087 |
|  | 0048011 |
|  | 0016032 |
|  | 0006184 |
|  | 0006915 |
|  | 0008624 |
|  | 0030155 |
|  | 0005624 |
|  | 0023034 |
|  | 0005515 |
|  | 0050775 |

---

|  |  |
| --- | --- |
| KEGG ID | hsa:5879 |
|  | mmu:19353 |
|  | rno:363875 |

---

|  |  |
| --- | --- |
| MedLine Reference | 11756405 |
|  | 11950936 |
|  | 12105187 |
|  | 12132588 |
|  | 12672819 |
|  | 12699088 |
|  | 12719791 |
|  | 12777392 |
|  | 12782387 |
|  | 14570905 |
|  | 14736704 |
|  | 15037605 |
|  | 15210825 |
|  | 10350213 |
|  | 12642504 |
|  | 15142861 |
|  | 15213258 |
|  | 15226395 |
|  | 15262848 |
|  | 15308668 |
|  | 15308673 |
|  | 15351707 |
|  | 15494521 |
|  | 15608632 |
|  | 15611338 |
|  | 15629443 |
|  | 15634677 |
|  | 15826947 |
|  | 16040606 |
|  | 16092944 |
|  | 16168664 |
|  | 16283857 |
|  | 16291935 |
|  | 16354758 |
|  | 16439689 |
|  | 16515795 |
|  | 16651530 |
|  | 16698001 |
|  | 16801538 |
|  | 16954223 |
|  | 17088251 |
|  | 17109063 |
|  | 17109064 |
|  | 17130476 |
|  | 10030666 |
|  | 10349636 |
|  | 10464095 |
|  | 10699171 |
|  | 10725249 |
|  | 10821761 |
|  | 10922068 |
|  | 11042159 |
|  | 11076861 |
|  | 11217851 |
|  | 11713255 |
|  | 11728333 |
|  | 11747086 |
|  | 11790798 |
|  | 11884408 |
|  | 11923206 |
|  | 12213441 |
|  | 12351724 |
|  | 12376551 |
|  | 12384416 |
|  | 12391220 |
|  | 12446704 |
|  | 12466851 |
|  | 12477932 |
|  | 12482591 |
|  | 12499359 |
|  | 12525493 |
|  | 12566226 |
|  | 12578823 |
|  | 12617831 |
|  | 12759446 |
|  | 12783890 |
|  | 12805377 |
|  | 12810717 |
|  | 12829743 |
|  | 12853475 |
|  | 12857864 |
|  | 12861025 |
|  | 12874273 |
|  | 12912917 |
|  | 14506233 |
|  | 14516655 |
|  | 14564009 |
|  | 14564011 |
|  | 14573547 |
|  | 14575316 |
|  | 14580336 |
|  | 14594451 |
|  | 14622142 |
|  | 14657280 |
|  | 14697354 |
|  | 14701795 |
|  | 14718541 |
|  | 14764880 |
|  | 14996945 |
|  | 15054110 |
|  | 15060067 |
|  | 15102830 |
|  | 15151987 |
|  | 15234347 |
|  | 15249579 |
|  | 15265981 |
|  | 15271660 |
|  | 15292195 |
|  | 15308574 |
|  | 15326354 |
|  | 15371457 |
|  | 15383465 |
|  | 15388231 |
|  | 15466361 |
|  | 15504745 |
|  | 15528331 |
|  | 15557338 |
|  | 15579468 |
|  | 15647276 |
|  | 15703194 |
|  | 15728191 |
|  | 15728722 |
|  | 15782199 |
|  | 15883643 |
|  | 15893749 |
|  | 15964829 |
|  | 16002401 |
|  | 16025125 |
|  | 16055737 |
|  | 16081735 |
|  | 16081809 |
|  | 16141072 |
|  | 16141073 |
|  | 16155095 |
|  | 16157315 |
|  | 16170364 |
|  | 16186118 |
|  | 16195235 |
|  | 16294277 |
|  | 16362953 |
|  | 16440308 |
|  | 16462764 |
|  | 16521128 |
|  | 16525021 |
|  | 16525721 |
|  | 16546099 |
|  | 16599904 |
|  | 16624865 |
|  | 16690620 |
|  | 16698790 |
|  | 16754961 |
|  | 16802292 |
|  | 16809619 |
|  | 16814769 |
|  | 16863904 |
|  | 16882712 |
|  | 16917494 |
|  | 16943436 |
|  | 17012749 |
|  | 17021167 |
|  | 17041601 |
|  | 17041603 |
|  | 1905006 |
|  | 7671812 |
|  | 7888179 |
|  | 8543060 |
|  | 9395237 |
|  | 9438836 |
|  | 10051605 |
|  | 10220378 |
|  | 10346909 |
|  | 10486263 |
|  | 10508610 |
|  | 10523675 |
|  | 10559471 |
|  | 10597294 |
|  | 10617634 |
|  | 10673424 |
|  | 10747961 |
|  | 10766742 |
|  | 10779525 |
|  | 10816584 |
|  | 10871853 |
|  | 10954424 |
|  | 10958683 |
|  | 11021801 |
|  | 11027608 |
|  | 11030651 |
|  | 11062023 |
|  | 11084340 |
|  | 11090627 |
|  | 11101877 |
|  | 11130063 |
|  | 11130076 |
|  | 11134022 |
|  | 11258927 |
|  | 11260256 |
|  | 11278572 |
|  | 11284700 |
|  | 11416133 |
|  | 11422783 |
|  | 11438723 |
|  | 11457725 |
|  | 11478794 |
|  | 11513579 |
|  | 11590143 |
|  | 11595749 |
|  | 11685227 |
|  | 11756552 |
|  | 11822867 |
|  | 11839748 |
|  | 11884513 |
|  | 11893732 |
|  | 11896062 |
|  | 11948427 |
|  | 11950930 |
|  | 11973651 |
|  | 11998687 |
|  | 12006387 |
|  | 12027902 |
|  | 12038964 |
|  | 12054568 |
|  | 12086876 |
|  | 12115629 |
|  | 12117911 |
|  | 12123608 |
|  | 12134158 |
|  | 12169092 |
|  | 12181570 |
|  | 12239215 |
|  | 12377770 |
|  | 12393431 |
|  | 12393632 |
|  | 12453877 |
|  | 12499846 |
|  | 12511425 |
|  | 12524240 |
|  | 12533515 |
|  | 12547822 |
|  | 12574162 |
|  | 12584113 |
|  | 12606638 |
|  | 12612085 |
|  | 12650940 |
|  | 12657628 |
|  | 12676940 |
|  | 12690205 |
|  | 12714565 |
|  | 12716910 |
|  | 12719789 |
|  | 12761036 |
|  | 12853948 |
|  | 12879077 |
|  | 12883706 |
|  | 12912997 |
|  | 12915445 |
|  | 12919680 |
|  | 12960245 |
|  | 12972426 |
|  | 14514689 |
|  | 14557270 |
|  | 14576080 |
|  | 14623895 |
|  | 14625275 |
|  | 14641180 |
|  | 14702039 |
|  | 14729058 |
|  | 14736526 |
|  | 14737186 |
|  | 14960575 |
|  | 14976202 |
|  | 15009099 |
|  | 15023524 |
|  | 15048733 |
|  | 15121898 |
|  | 15123640 |
|  | 15123662 |
|  | 15166228 |
|  | 15170282 |
|  | 15187145 |
|  | 15194684 |
|  | 15194790 |
|  | 15226424 |
|  | 15231571 |
|  | 15247305 |
|  | 15265891 |
|  | 15298851 |
|  | 15303276 |
|  | 15304341 |
|  | 15377999 |
|  | 15470041 |
|  | 15535843 |
|  | 15569826 |
|  | 15614286 |
|  | 15777850 |
|  | 15817154 |
|  | 15850775 |
|  | 15860737 |
|  | 15864742 |
|  | 15901767 |
|  | 15905615 |
|  | 15976327 |
|  | 16027728 |
|  | 16195373 |
|  | 16243562 |
|  | 16246732 |
|  | 16257181 |
|  | 16280046 |
|  | 16339170 |
|  | 16339173 |
|  | 16354686 |
|  | 16390331 |
|  | 16395716 |
|  | 16397238 |
|  | 16410245 |
|  | 16492808 |
|  | 16507994 |
|  | 16551621 |
|  | 16628223 |
|  | 16681996 |
|  | 16707128 |
|  | 16716260 |
|  | 16751412 |
|  | 16762923 |
|  | 16772332 |
|  | 16775008 |
|  | 16825658 |
|  | 16861229 |
|  | 16914518 |
|  | 17027967 |
|  | 17064663 |
|  | 17074883 |
|  | 17115053 |
|  | 2108320 |
|  | 2674130 |
|  | 7493928 |
|  | 7545969 |
|  | 7629060 |
|  | 7673236 |
|  | 7738010 |
|  | 7744004 |
|  | 7938008 |
|  | 7957046 |
|  | 8034624 |
|  | 8036496 |
|  | 8107774 |
|  | 8491184 |
|  | 8496137 |
|  | 8631991 |
|  | 8643625 |
|  | 8670801 |
|  | 8670882 |
|  | 8702968 |
|  | 8756646 |
|  | 8769096 |
|  | 8798539 |
|  | 9083043 |
|  | 9148763 |
|  | 9255350 |
|  | 9305638 |
|  | 9312003 |
|  | 9417078 |
|  | 9427749 |
|  | 9463375 |
|  | 9535855 |
|  | 9572733 |
|  | 9582072 |
|  | 9624165 |
|  | 9786927 |
|  | 9799233 |
|  | 9808620 |
|  | 9843499 |
|  | 9857026 |
|  | 11035813 |
|  | 11135665 |
|  | 11163217 |
|  | 11346801 |
|  | 11513578 |
|  | 11955434 |
|  | 15489334 |
|  | 1643658 |
|  | 16982419 |
|  | 17081065 |
|  | 17121847 |
|  | 17122362 |
|  | 17229424 |
|  | 17233629 |
|  | 17234718 |
|  | 17244648 |
|  | 17275330 |
|  | 17300916 |
|  | 17301947 |
|  | 17347137 |
|  | 17356711 |
|  | 17409253 |
|  | 17428306 |
|  | 17478078 |
|  | 17513457 |
|  | 17517067 |
|  | 17562712 |
|  | 17568564 |
|  | 17622308 |
|  | 17663742 |
|  | 1903399 |
|  | 8990187 |
|  | 9030878 |
|  | 9033596 |
|  | 9190911 |
|  | 10934474 |
|  | 10934475 |
|  | 14634666 |
|  | 16195887 |
|  | 16990606 |
|  | 17090688 |
|  | 17115888 |
|  | 17178910 |
|  | 17265067 |
|  | 17267693 |
|  | 17276347 |
|  | 17301832 |
|  | 17332503 |
|  | 17334398 |
|  | 17389689 |
|  | 17409386 |
|  | 17428196 |
|  | 17467682 |
|  | 17573353 |
|  | 17576798 |
|  | 17576799 |
|  | 17653061 |
|  | 17804720 |
|  | 7561098 |
|  | 15721239 |
|  | 15723051 |
|  | 17085464 |
|  | 17183546 |
|  | 17207463 |
|  | 17515454 |
|  | 17515837 |
|  | 17687038 |

---

|  |  |
| --- | --- |
| Organism | Homo sapiens {Organism urn:agi-taxid:9606} |
|  | Mus musculus {Organism urn:agi-taxid:10090} |
|  | Rattus norvegicus {Organism urn:agi-taxid:10116} |
|  | Homo sapiens |
|  | Mus musculus |
|  | Rattus norvegicus |

---

|  |  |
| --- | --- |
| Mouse chromosome position | 5 82.22 cM |
|  | 5 82.0 cM |

---

|  |  |
| --- | --- |
| OMIM ID | 602048 |
|  | 617751 |

---

|  |  |
| --- | --- |
| Rat chromosome position | 12p11 |

---

|  |  |
| --- | --- |
| Hugo ID | 9801 |
|  | HGNC:9801 |

---

|  |  |
| --- | --- |
| Human chromosome position | 7p22.1 |
|  | 7p22 |

---

|  |  |
| --- | --- |
| Swiss-Prot Accession | A4D2P1 |
|  | P63000 |
|  | A4D2P0 |
|  | P63000.1 |
|  | Q3TLP8 |
|  | K7Q7T7 |
|  | P63001 |
|  | P63001.1 |
|  | Q6RUV5 |
|  | Q6RUV5.1 |
|  | O95501 |
|  | P15154 |
|  | Q3Y4D3 |
|  | Q5JAA8 |
|  | Q9BTB4 |
|  | Q59FQ0 |
|  | Q8BPG5 |
|  | Q8C4N8 |
|  | Q9D859 |
|  | Q923X0 |
|  | AAB22206 |
|  | RAC1\_HUMAN |
|  | RAC1\_MOUSE |
|  | RAC1\_RAT |
|  | A4D2P0\_HUMAN |
|  | A4D2P1\_HUMAN |

---

|  |  |
| --- | --- |
| PIR ID | A34788 |
|  | A60347 |

---

|  |  |
| --- | --- |
| GenBank ID | NC\_000007 |
|  | NM\_018890 |
|  | NP\_061485 |
|  | NM\_006908 |
|  | NP\_008839 |
|  | NG\_029431 |
|  | AC009412 |
|  | AAS07511 |
|  | AAS07512 |
|  | AJ132695 |
|  | CAA10733 |
|  | CAB53579 |
|  | AL833528 |
|  | CH236963 |
|  | EAL23718 |
|  | EAL23719 |
|  | EAL23720 |
|  | CH878731 |
|  | EAW55039 |
|  | EAW55040 |
|  | EAW55041 |
|  | EAW55042 |
|  | EAW55043 |
|  | EAW55044 |
|  | CS026591 |
|  | CAI61762 |
|  | CS026817 |
|  | CAI61874 |
|  | DQ165078 |
|  | AAZ80485 |
|  | AB209410 |
|  | BAD92647 |
|  | AB451238 |
|  | BAG70052 |
|  | AB451363 |
|  | BAG70177 |
|  | AF136373 |
|  | AAD30547 |
|  | AF498964 |
|  | AAM21111 |
|  | AJ132694 |
|  | CAA10732 |
|  | AK054993 |
|  | BAG51447 |
|  | AK122742 |
|  | AK291806 |
|  | BAF84495 |
|  | AK291815 |
|  | BAF84504 |
|  | AY279384 |
|  | AAQ16632 |
|  | BC004247 |
|  | AAH04247 |
|  | BC050687 |
|  | AAH50687 |
|  | BC107748 |
|  | AAI07749 |
|  | BG437715 |
|  | BQ689587 |
|  | BT007121 |
|  | AAP35785 |
|  | D25274 |
|  | M29870 |
|  | AAA36537 |
|  | M31467 |
|  | AAA36544 |
|  | P63000 |
|  | NC\_000071 |
|  | XM\_006504667 |
|  | XP\_006504730 |
|  | NM\_009007 |
|  | NP\_033033 |
|  | NM\_001347530 |
|  | AC163995 |
|  | AC175091 |
|  | AC217112 |
|  | CH466529 |
|  | EDL19046 |
|  | EDL19047 |
|  | EDL19048 |
|  | AK008435 |
|  | BAB25667 |
|  | AK009017 |
|  | BAB26027 |
|  | AK011072 |
|  | BAB69451 |
|  | AK034601 |
|  | BAC28767 |
|  | AK047969 |
|  | BAC33203 |
|  | AK076023 |
|  | BAC36128 |
|  | AK081613 |
|  | BAC38272 |
|  | AK088825 |
|  | BAC40596 |
|  | AK166383 |
|  | BAE38744 |
|  | AK181319 |
|  | AK182603 |
|  | AK184868 |
|  | AK186906 |
|  | AK190884 |
|  | AK199407 |
|  | AK209867 |
|  | AK211591 |
|  | AK216533 |
|  | AK219413 |
|  | BC003828 |
|  | AAH03828 |
|  | BC051053 |
|  | AAH51053 |
|  | BQ444803 |
|  | CO425367 |
|  | CX242948 |
|  | CX566679 |
|  | JN971018 |
|  | AFR23587 |
|  | U25107 |
|  | AAB42023 |
|  | X57277 |
|  | CAA40545 |
|  | P63001 |
|  | NC\_005111 |
|  | XM\_008768986 |
|  | XP\_008767208 |
|  | NM\_134366 |
|  | NP\_599193 |
|  | AC\_000080 |
|  | AABR07035397 |
|  | AAHX01071257 |
|  | CH474012 |
|  | EDL89664 |
|  | EDL89665 |
|  | EDL89666 |
|  | AF385833 |
|  | AAK66567 |
|  | AY491395 |
|  | AAR84574 |
|  | FQ209708 |
|  | FQ230945 |
|  | Q6RUV5 |
|  | NC\_018918 |
|  | AMYH02015172 |
|  | AMYH02015173 |
|  | AAB22206 |
|  | EU831965 |
|  | ACE87406 |
|  | EU832058 |
|  | ACE86720 |
|  | EU832640 |
|  | ACE87731 |
|  | EU832717 |
|  | ACE87049 |
|  | AC\_000027 |
|  | AAHY01050472 |
|  | AAHY01050473 |
|  | AC\_000139 |
|  | ABBA01056484 |
|  | ABBA01056485 |
|  | AC\_000068 |
|  | AACC02000121 |
|  | AABR06070505 |
|  | NT\_007819 |
|  | NT\_079592 |
|  | NW\_923217 |
|  | NW\_001839003 |
|  | AC\_000050 |
|  | CR591797 |
|  | CR612844 |
|  | CR621312 |
|  | CR624356 |
|  | CR624866 |
|  | Q59FQ0 |
|  | NT\_081055 |
|  | NW\_001030796 |
|  | Q3TLP8 |
|  | Q8BPG5 |
|  | Q8C4N8 |
|  | Q9D859 |
|  | NW\_001084671 |
|  | NW\_047368 |
|  | Q923X0 |
|  | AK195884 |
|  | NM\_198829 |
|  | NP\_942126 |
|  | AK193118 |
|  | BG064327 |
|  | Y12699 |
|  | U80083 |
|  | Q5JAA8 |
|  | AL023026 |
|  | U77544 |
|  | U85236 |
|  | AAB36753 |
|  | AAC50998 |
|  | U91215 |
|  | BG088950 |
|  | CAA73231 |
|  | AAC51432 |
|  | AAB58438 |
|  | NW\_000241 |
|  | BG076502 |
|  | 14532301 |
|  | 40354188 |
|  | 51701705 |
|  | 81902670 |
|  | 123786781 |
|  | 12842616 |
|  | 12843555 |
|  | 13277918 |
|  | 15919905 |
|  | 1816498 |
|  | 26330057 |
|  | 26339064 |
|  | 26344958 |
|  | 26349265 |
|  | 26353932 |
|  | 29835222 |
|  | 51702788 |
|  | 53886 |
|  | 74147756 |
|  | 81874558 |
|  | 81881587 |
|  | 81898825 |
|  | AU022720 |
|  | 119575441 |
|  | 119575442 |
|  | 119575444 |
|  | 119575446 |
|  | 13279011 |
|  | 1685223 |
|  | 1791009 |
|  | 190824 |
|  | 190875 |
|  | 20379102 |
|  | 2145168 |
|  | 2239837 |
|  | 2266631 |
|  | 249582 |
|  | 29792302 |
|  | 30583081 |
|  | 33358321 |
|  | 41473432 |
|  | 41473433 |
|  | 4454211 |
|  | 4836769 |
|  | 51094459 |
|  | 51094460 |
|  | 51094461 |
|  | 51702787 |
|  | 60496663 |
|  | 60496888 |
|  | 73672821 |
|  | 74741595 |
|  | 78070394 |
|  | 8574038 |
|  | 8574039 |
|  | P15154 |
|  | 119575443 |
|  | 119575445 |
|  | 62088400 |
|  | 74707268 |
|  | 148687099 |
|  | 148687100 |
|  | 148687101 |
|  | 149034944 |
|  | 149034945 |
|  | 149034946 |

---

|  |  |
| --- | --- |
| Swiss-Prot ID | RAC1\_HUMAN |
|  | RAC1\_MOUSE |
|  | RAC1\_RAT |
|  | A4D2P1\_HUMAN |
|  | A4D2P0\_HUMAN |

---

|  |  |
| --- | --- |
| Cell Localization | Cell membrane |
|  | Melanosome |
|  | Cytoplasm |
|  | Cell projection |
|  | Lamellipodium |
|  | Dendrite |
|  | Plasma membrane |

---

|  |  |
| --- | --- |
| Ensembl ID | ENSG00000136238 |
|  | ENSP00000258737.7 |
|  | ENST00000348035.9 |
|  | ENSP00000348461.4 |
|  | ENST00000356142.4 |
|  | ENSMUSG00000001847 |
|  | ENSMUSP00000079380.7 |
|  | ENSMUST00000080537.13 |
|  | ENSMUSP00000098058.3 |
|  | ENSMUST00000100489.3 |
|  | ENSRNOG00000001068 |
|  | ENSRNOP00000001417.3 |
|  | ENSRNOT00000001417.5 |
|  | ENSRNOP00000071616.1 |
|  | ENSRNOT00000087094.1 |
|  | ENSP00000258737 |
|  | ENST00000348035 |
|  | ENSP00000348461 |
|  | ENST00000356142 |
|  | ENSMUSP00000079380 |
|  | ENSMUST00000080537 |
|  | ENSMUSP00000098058 |
|  | ENSMUST00000100489 |
|  | ENSRNOP00000001417 |
|  | ENSRNOT00000001417 |
|  | ENSRNOP00000071616 |
|  | ENSRNOT00000087094 |

---

|  |  |
| --- | --- |
| MGI ID | MGI:97845 |
|  | 97845 |
|  | 2141016 |
|  | 1261850 |

---

|  |  |
| --- | --- |
| RGD ID | 619755 |

---

|  |  |
| --- | --- |
| Unigene ID | Hs.413812 |
|  | Mm.292510 |
|  | Mm.469963 |
|  | Mm.475073 |
|  | Rn.29157 |
|  | Mm.342177 |
|  | Mm.466548 |
|  | Mm.74208 |
|  | Hs.632900 |
|  | Hs.633454 |
|  | Hs.551925 |
|  | Mm.432479 |

---

|  |  |
| --- | --- |
| Homologene ID | 69035 |

---

|  |  |
| --- | --- |
| IPI ID | IPI00010271 |
|  | IPI00219675 |
|  | IPI00422092 |
|  | IPI00761613 |
|  | IPI00555566 |
|  | IPI00394837 |
|  | IPI00127408 |
|  | IPI00780227 |

---

|  |  |
| --- | --- |
| Homo sapiens Chromosome position | 7p22 |

---

|  |  |
| --- | --- |
| Ensembl Transcript ID | ENST00000348035 |
|  | ENST00000356142 |
|  | ENST00000396729 |

---

|  |  |
| --- | --- |
| ENSEMBL | ENSG00000136238 |

---

|  |  |
| --- | --- |
| FunctionalClass | GTP binding |
|  | GTPase |
|  | protein binding |
|  | nucleotide binding |
|  | GTP-dependent protein binding |

---

|  |  |
| --- | --- |
| Mus musculus Chromosome position | 5 82.0 cM |
|  | 5 |

---

|  |  |
| --- | --- |
| ProteinAtlas SubcellularLocation | Main location: Nucleus;Cytoplasm; Other location: Nucleoli; Expression type: Staining; Reliability: Uncertain |

---

|  |  |
| --- | --- |
| Rattus norvegicus Chromosome position | 12p11 |

---

|  |  |
| --- | --- |
| Ensembl Gene ID | ENSG00000136238 |

---

|  |  |
| --- | --- |
| ProteinAtlas NormalTissue | Tissue: adrenal gland; Cell type: glandular cells; Level: Moderate; Expression type: Staining; Reliability: Supportive |
|  | Tissue: appendix; Cell type: glandular cells; Level: Moderate; Expression type: Staining; Reliability: Supportive |
|  | Tissue: appendix; Cell type: lymphoid tissue; Level: Negative; Expression type: Staining; Reliability: Supportive |
|  | Tissue: bone marrow; Cell type: hematopoietic cells; Level: Moderate; Expression type: Staining; Reliability: Supportive |
|  | Tissue: breast; Cell type: glandular cells; Level: Negative; Expression type: Staining; Reliability: Supportive |
|  | Tissue: bronchus; Cell type: respiratory epithelial cells; Level: Weak; Expression type: Staining; Reliability: Supportive |
|  | Tissue: cerebellum; Cell type: cells in granular layer; Level: Negative; Expression type: Staining; Reliability: Supportive |
|  | Tissue: cerebellum; Cell type: cells in molecular layer; Level: Negative; Expression type: Staining; Reliability: Supportive |
|  | Tissue: cerebellum; Cell type: Purkinje cells; Level: Weak; Expression type: Staining; Reliability: Supportive |
|  | Tissue: cerebral cortex; Cell type: glial cells; Level: Negative; Expression type: Staining; Reliability: Supportive |
|  | Tissue: cerebral cortex; Cell type: neuronal cells; Level: Negative; Expression type: Staining; Reliability: Supportive |
|  | Tissue: cervix, uterine; Cell type: glandular cells; Level: Negative; Expression type: Staining; Reliability: Supportive |
|  | Tissue: cervix, uterine; Cell type: squamous epithelial cells; Level: Weak; Expression type: Staining; Reliability: Supportive |
|  | Tissue: colon; Cell type: glandular cells; Level: Strong; Expression type: Staining; Reliability: Supportive |
|  | Tissue: duodenum; Cell type: glandular cells; Level: Moderate; Expression type: Staining; Reliability: Supportive |
|  | Tissue: epididymis; Cell type: glandular cells; Level: Negative; Expression type: Staining; Reliability: Supportive |
|  | Tissue: esophagus; Cell type: squamous epithelial cells; Level: Negative; Expression type: Staining; Reliability: Supportive |
|  | Tissue: fallopian tube; Cell type: glandular cells; Level: Moderate; Expression type: Staining; Reliability: Supportive |
|  | Tissue: gall bladder; Cell type: glandular cells; Level: Moderate; Expression type: Staining; Reliability: Supportive |
|  | Tissue: heart muscle; Cell type: myocytes; Level: Negative; Expression type: Staining; Reliability: Supportive |
|  | Tissue: hippocampus; Cell type: glial cells; Level: Negative; Expression type: Staining; Reliability: Supportive |
|  | Tissue: hippocampus; Cell type: neuronal cells; Level: Negative; Expression type: Staining; Reliability: Supportive |
|  | Tissue: kidney; Cell type: cells in glomeruli; Level: Negative; Expression type: Staining; Reliability: Supportive |
|  | Tissue: kidney; Cell type: cells in tubules; Level: Weak; Expression type: Staining; Reliability: Supportive |
|  | Tissue: lateral ventricle; Cell type: glial cells; Level: Negative; Expression type: Staining; Reliability: Supportive |
|  | Tissue: lateral ventricle; Cell type: neuronal cells; Level: Negative; Expression type: Staining; Reliability: Supportive |
|  | Tissue: liver; Cell type: bile duct cells; Level: Negative; Expression type: Staining; Reliability: Supportive |
|  | Tissue: liver; Cell type: hepatocytes; Level: Negative; Expression type: Staining; Reliability: Supportive |
|  | Tissue: lung; Cell type: macrophages; Level: Negative; Expression type: Staining; Reliability: Supportive |
|  | Tissue: lung; Cell type: pneumocytes; Level: Moderate; Expression type: Staining; Reliability: Supportive |
|  | Tissue: lymph node; Cell type: germinal center cells; Level: Negative; Expression type: Staining; Reliability: Supportive |
|  | Tissue: lymph node; Cell type: non-germinal center cells; Level: Negative; Expression type: Staining; Reliability: Supportive |
|  | Tissue: nasopharynx; Cell type: respiratory epithelial cells; Level: Moderate; Expression type: Staining; Reliability: Supportive |
|  | Tissue: oral mucosa; Cell type: squamous epithelial cells; Level: Negative; Expression type: Staining; Reliability: Supportive |
|  | Tissue: ovary; Cell type: ovarian stroma cells; Level: Negative; Expression type: Staining; Reliability: Supportive |
|  | Tissue: pancreas; Cell type: exocrine glandular cells; Level: Weak; Expression type: Staining; Reliability: Supportive |
|  | Tissue: pancreas; Cell type: islets of Langerhans; Level: Negative; Expression type: Staining; Reliability: Supportive |
|  | Tissue: parathyroid gland; Cell type: glandular cells; Level: Negative; Expression type: Staining; Reliability: Supportive |
|  | Tissue: placenta; Cell type: decidual cells; Level: Moderate; Expression type: Staining; Reliability: Supportive |
|  | Tissue: placenta; Cell type: trophoblastic cells; Level: Negative; Expression type: Staining; Reliability: Supportive |
|  | Tissue: prostate; Cell type: glandular cells; Level: Weak; Expression type: Staining; Reliability: Supportive |
|  | Tissue: rectum; Cell type: glandular cells; Level: Moderate; Expression type: Staining; Reliability: Supportive |
|  | Tissue: salivary gland; Cell type: glandular cells; Level: Negative; Expression type: Staining; Reliability: Supportive |
|  | Tissue: seminal vesicle; Cell type: glandular cells; Level: Negative; Expression type: Staining; Reliability: Supportive |
|  | Tissue: skeletal muscle; Cell type: myocytes; Level: Weak; Expression type: Staining; Reliability: Supportive |
|  | Tissue: skin; Cell type: epidermal cells; Level: Weak; Expression type: Staining; Reliability: Supportive |
|  | Tissue: small intestine; Cell type: glandular cells; Level: Moderate; Expression type: Staining; Reliability: Supportive |
|  | Tissue: smooth muscle; Cell type: smooth muscle cells; Level: Negative; Expression type: Staining; Reliability: Supportive |
|  | Tissue: spleen; Cell type: cells in red pulp; Level: Negative; Expression type: Staining; Reliability: Supportive |
|  | Tissue: spleen; Cell type: cells in white pulp; Level: Negative; Expression type: Staining; Reliability: Supportive |
|  | Tissue: stomach, lower; Cell type: glandular cells; Level: Weak; Expression type: Staining; Reliability: Supportive |
|  | Tissue: stomach, upper; Cell type: glandular cells; Level: Moderate; Expression type: Staining; Reliability: Supportive |
|  | Tissue: testis; Cell type: cells in seminiferus ducts; Level: Negative; Expression type: Staining; Reliability: Supportive |
|  | Tissue: testis; Cell type: Leydig cells; Level: Weak; Expression type: Staining; Reliability: Supportive |
|  | Tissue: thyroid gland; Cell type: glandular cells; Level: Negative; Expression type: Staining; Reliability: Supportive |
|  | Tissue: tonsil; Cell type: germinal center cells; Level: Negative; Expression type: Staining; Reliability: Supportive |
|  | Tissue: tonsil; Cell type: non-germinal center cells; Level: Negative; Expression type: Staining; Reliability: Supportive |
|  | Tissue: tonsil; Cell type: squamous epithelial cells; Level: Moderate; Expression type: Staining; Reliability: Supportive |
|  | Tissue: urinary bladder; Cell type: urothelial cells; Level: Moderate; Expression type: Staining; Reliability: Supportive |
|  | Tissue: uterus, post-menopause; Cell type: cells in endometrial stroma; Level: Negative; Expression type: Staining; Reliability: Supportive |
|  | Tissue: uterus, post-menopause; Cell type: glandular cells; Level: Weak; Expression type: Staining; Reliability: Supportive |
|  | Tissue: uterus, pre-menopause; Cell type: cells in endometrial stroma; Level: Negative; Expression type: Staining; Reliability: Supportive |
|  | Tissue: uterus, pre-menopause; Cell type: glandular cells; Level: Moderate; Expression type: Staining; Reliability: Supportive |
|  | Tissue: vagina; Cell type: squamous epithelial cells; Level: Negative; Expression type: Staining; Reliability: Supportive |
|  | Tissue: vulva/anal skin; Cell type: epidermal cells; Level: Negative; Expression type: Staining; Reliability: Supportive |

---

|  |  |
| --- | --- |
| LocatorString | digtext:C:\Users\Ariadne\Customers\Elan\Elan weekly report from literature condensed.txt |

---
